# Programs Addressing Food Security for First Nations Peoples: A Scoping Review

**DOI:** 10.3390/nu15143127

**Published:** 2023-07-13

**Authors:** Alyse Davies, Josephine Gwynn, Margaret Allman-Farinelli, Victoria Flood, Michelle Dickson, Nicole Turner, Bobby Porykali, Mark Lock (Ngiyampaa)

**Affiliations:** 1 Discipline of Nutrition and Dietetics, Susan Wakil School of Nursing and Midwifery, Faculty of Medicine and Health, The University of Sydney, Sydney, NSW 2006, Australia; margaret.allman-farinelli@sydney.edu.au; 2 Charles Perkins Centre, The University of Sydney, Sydney, NSW 2006, Australia; josephine.gwynn@sydney.edu.au (J.G.); nturner@nswrdn.com.au (N.T.); 3 Sydney School of Health Sciences, Faculty of Medicine and Health, The University of Sydney, Sydney, NSW 2006, Australia; vicki.flood@sydney.edu.au; 4 University Centre for Rural Health, Northern Rivers, Faculty of Medicine and Health, The University of Sydney, Lismore, NSW, 2480, Australia; 5 The Poche Centre for Indigenous Health, Faculty of Medicine and Health, The University of Sydney, Sydney, NSW 2006, Australia; michelle.dickson@sydney.edu.au; 6 Aboriginal and Torres Strait Islander Health Program, George Institute for Global Health, Sydney, NSW 2042, Australia; bporykali@georgeinstitute.org.au; 7 Global Centre for Preventive Health and Nutrition, Institute for Health Transformation, School of Health and Social Development, Faculty of Health, Deakin University, Melbourne, VIC 3220, Australia; mark.lock@deakin.edu.au

**Keywords:** First Nations, food security, food insecurity

## Abstract

Access to food is a right that every individual must have to ensure a standard of living that is sufficient for maintaining good health and wellbeing. This review, developed and implemented by a team of First Nations and non-First Nations peoples, aimed to scope the literature on programs addressing food security for First Nations peoples in Australia, Aotearoa/New Zealand, Canada, and the United States of America. Collectively, First Nations groups share continued traumas, disadvantages, and devastation brought upon them as a result of British colonisation. Despite the impacts of colonial conquest, the resilience of First Nations peoples continues through the fight for self-determination, sovereignty, equity, and equality. Three databases and grey literature were searched from 2010. Two reviewers completed screening, data extraction, and critical appraisal. Nine food security programs were included in this review. Five were from the United States of America and four from Canada, with no program from Australia or Aotearoa/New Zealand meeting the inclusion criteria. The programs that appear to be most suitable for addressing food security for First Nations peoples were participatory in design, had community governance, integrated cultural knowledge and food systems to increase the accessibility and availability of cultural foods, incorporated educational components, and utilized collaborations among various agencies. Findings showed that while it is important to address short-term emergency food relief, the aim should be sustainable food security through a longer-term system and policy change underpinned by co-designed research and evaluation.

## 1. Introduction

The deep and interconnected relationship of First Nations peoples of Australia, Aotearoa/New Zealand, Canada, and the United States of America to their lands, waterways, and seas ensured optimum health, and cultural, spiritual, social, and emotional wellbeing. For thousands of years, advanced agriculture and aquacultural techniques supplied ample and dependable abundance of fresh and nutritious foods, with the guiding principle behind sustainable harvesting and food procurement practices being the care for ‘Country’ [1]. Though proudly representing distinct cultures, traditions, values, and lands, collectively, First Nation groups share continued traumas, disadvantages, and devastation brought upon them because of British colonisation in the 1600s, in 1788, and in the 1800s, respectively, for North America (or what would become Canada and the United States of America), Australia, and Aotearoa/New Zealand. The forceful disconnect from land, waterways, seas, culture, and knowledge systems has reshaped the availability and accessibility to foods, food procurement practices, and relationship to cultural food systems. The lasting impacts of colonisation contribute to the collective social, economic, and systemic disparities which serve as significant underlying factors affecting food security. 

In the literature, many definitions exist for food security. The Food and Agriculture Organization (FAO) of the United Nations defines food security as “When all people, at all times, have physical and economic access to sufficient, safe and nutritious food to meet their dietary needs and food preferences for an active and healthy life” [2]. Building on the FAO definition, a more holistic definition was developed by Aboriginal peoples in the Northern Territory of Australia through the Menzies School of Health in order to incorporate the importance of culture and traditional foods “The land and the sea is our food security. It is our right. Food security for us has two parts: Food security is when the food from our ancestors is protected and always there for us and our children. It is also when we can easily access and afford the right non-traditional food for a collective healthy and active life. When we are food secure, we can provide, share and fulfil our responsibilities, we can choose good food, knowing how to make choices and how to prepare and use it” [3]. Food security can be measured within the household or at a community or population level. The four pillars of food security include availability, accessibility, utilization, and stability [4,5], and as agency and sustainability relate to food insecurity, they have also been recognised as important additional dimensions [6].

Access to food is a right that every individual must have to ensure a standard of living that is sufficient for maintaining good health and wellbeing [7]. In Australia, estimates of food insecurity for Aboriginal and Torres Strait Islander peoples range from 22 to 32% [8]. A recent report where Aboriginal community members were interviewed from a rural town in New South Wales (NSW) showed that 46% experience food insecurity [9]. While rates of food insecurity are highest in remote communities, those living in urban and regional areas are also vulnerable [10]. In Aotearoa/New Zealand, food insecurity is a major public health issue for Māori people [11]. National health data from 2015 to 2016 showed that the prevalence of food insecurity for the Māori population was 29%, with 19% of children living in households that experience moderate to severe food insecurity [12]. In Canada, the First Nations Regional Health Survey showed that food insecurity was experienced by 54% of households, while the Inuit Health Survey reported a range between 45 and 69% depending on the region [13,14]. Within the United States of America, the weighted prevalence of food insecurity among American Indians and Alaska Native peoples is 46%, although estimates ranged from 16 to 80% [15]. The contemporary pressures of earning an income and providing for one’s family or community, coupled with the broader stressors beyond monetary constraints linked to historical trauma, changing socio-cultural systems, and acculturative stress, increases vulnerability to food insecurity. Improving food security is a key aspect of Australia’s National Aboriginal and Torres Strait Islander Health Plan 2021–31 [16], and a key action area in Food Policy for Canada [17], but Aotearoa/New Zealand is lacking a food security policy [18], and it has been documented that a complex environmental approach is needed in order to enhance food security [19]. 

Food assistance programs have been developed to support individuals or families facing food insecurity to improve access to healthy foods. In the United States of America, the Supplemental Nutrition Assistance Program (SNAP), formerly known as Food Stamps, is the largest federal government food relief program. Others include the Special Supplemental Nutrition Program for Women, Infants, and Children (WIC), and the United States Department of Agriculture (USDA) Food Distribution Program on Indian Reservations (FDPIR). The views of residents of the Flathead Indian Reservations about food environments and nutrition was documented by Shanks et al. [20], and the recipients of SNAP and WIC reported that food assistance was helpful and contributed to food security. Australia has no government-funded national food-assistance program [21]. Whilst First Nations peoples of Australia, Aotearoa/New Zealand, Canada, and the United States of America experience the ongoing impacts of colonial conquest, they equally share in their continued fight for self-determination, sovereignty, equity, and equality. We asked: what lessons can be learned from food security initiatives worldwide that provide guidance for food security programs in Australia? This scoping review aims to assess the literature on programs effectively addressing food security for First Nations peoples in Australia, Aotearoa/New Zealand, Canada, and the United States of America. Throughout this review, we respectfully use the term First Nations when reporting on First Nations peoples of Australia, Aotearoa/New Zealand, Canada, and the United States of America.

## 2. Materials and Methods

### 2.1. Review Team and Protocol

This review team comprised First Nations (M.D., N.T., B.P., M.L.) and non-First Nations (A.D., J.G., M.A.-F., V.F.) with extensive expertise in nutrition, dietetics, and First Nations public health research. The researchers’ approach allowed for the situated knowledge of each First Nations reviewer to be reflected, and for a decolonised lens to be applied in each stage of this review process [22,23].

An a priori protocol was developed in collaboration with the researchers, Sax Institute, and Aboriginal Affairs NSW for a rapid review published on the Aboriginal Affairs website on 1 November 2022 [1]. The scoping review findings are reported in line with methodologies for scoping reviews [24,25].

### 2.2. Inclusion Criteria

#### 2.2.1. Participants

The included populations were First Nations peoples of Australia, Aotearoa/New Zealand, Canada, and the United States of America (inclusive of studies reporting data from First Nations and non-First Nations peoples).

#### 2.2.2. Concept

This review considered programs or interventions addressing food security and food insecurity. Programs were excluded if they indirectly addressed food (in)security, such as increasing the access to fruit and vegetables, evaluating the food environment, or exploring food availability and consumption of traditional foods. Studies documenting users of food assistance programs were also excluded.

#### 2.2.3. Context

This review considered any region, state, territory, province, nation, tribal land, or reservation of Australia, Aotearoa/New Zealand, Canada, and the United States of America (excluding South America, Central America, Latin America, and Mexico).

### 2.3. Types of Studies

The original protocol had a ten-year time period, but initial searches identified relevant articles from 2010; therefore, the time period from 2010 was applied. All study designs from peer-reviewed and grey literature were included except posters and abstracts, theses, editorials, newsletters, media, book chapters, commentaries, dissertations, protocols, conference proceedings, reviews, and meta-analysis.

### 2.4. Search Strategy

Two reviewers (A.D., M.L.) of which one was First Nations, developed the search strategy with an experienced librarian (M.C.) and agreement was reached with the review team. Three electronic databases were searched: Medline, Global Health, and Scopus. The MEDLINE search strategy is provided in Supplementary Materials Table S1.

Additionally, grey literature sources were searched. In Google Scholar, government domain names were used. Other relevant sources included the Australian Indigenous HealthInfoNet, Analysis and Policy Observatory, and The Conversation. A preliminary literature review was provided for the rapid review, and the reference list was searched, as was the Appendix of the rapid review project proposal.

### 2.5. Selection Process

**Figure 1:**
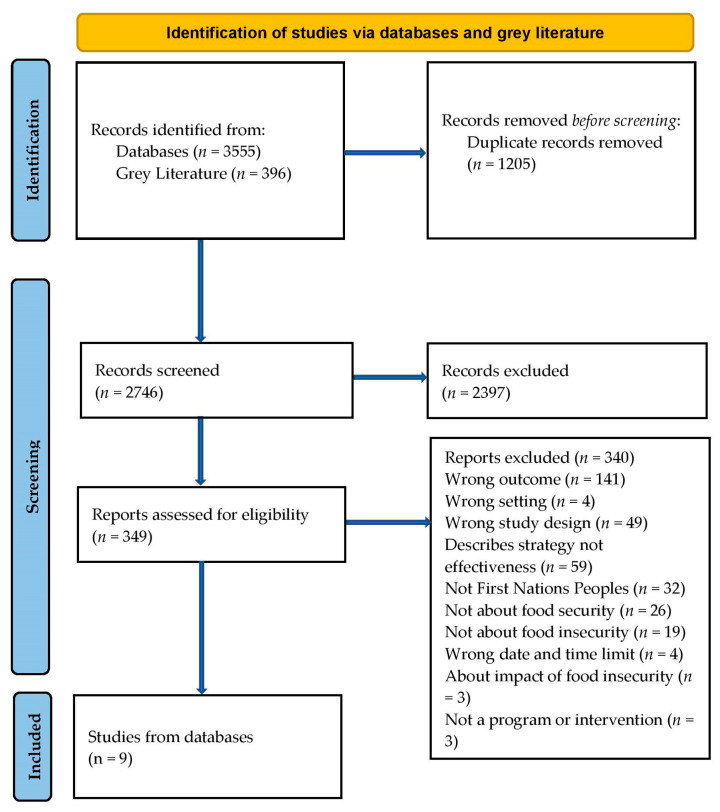
PRISMA flow diagram for the programs addressing food security for First Nations peoples of Australia, Aotearoa/New Zealand, Canada, and the United States of America.

The records were transferred into EndNote 20 (Clarivate Analytics, Philadelphia, PA, USA). The citations were imported into Covidence (Veritas Health Innovation, Melbourne, Australia). Duplicates were removed in both steps. Title and abstract screening were assessed by one reviewer (A.D.). Eligibility of the full text were assessed by two reviewers (J.G., M.L.) of which one was First Nations. The reasons for exclusion were recorded in Covidence. The search results are presented in a PRISMA flow diagram (see Figure 1).

### 2.6. Data Extraction and Charting

The following information was extracted according to the JBI Reviewer Manual by one reviewer (A.D.) and checked by another (M.A.-F.): study information (author, year, country, and aims); study characteristics (population and sample size); methods; intervention; and key findings. A narrative summary describes the results.

### 2.7. Critical Appraisal

Given First Nations peoples of Australia, Aotearoa/New Zealand, Canada, and the United States of America share similar colonial history of food system disruption, there is a shared purpose for self-determination, participatory community-based research, culturally appropriate programs, and food sovereignty. In line with decolonising research, the Aboriginal and Torres Strait Islander Quality Appraisal Tool (QAT) was developed for use in reviewing studies about First Nations Australians [26] and a companion document to guide appraisal [27]. We were cognizant of the cultural differences between nations included in this review and, following discussion, agreed that the 14 items in the QAT could be applied here given the shared colonial history. The tool assesses the quality of studies from a decolonised lens including First Nations governance, respect for cultural and intellectual property, capacity building, and beneficial outcomes. Using explicit statements in the text, two reviewers (M.A.-F., V.F.) selected from four options for each question; “yes, partially, no and unclear” with oversight by First Nations reviewers (M.D., M.L.). If “yes or partially” was documented for ten questions or more, the study was considered high quality, moderate for six to nine questions, and low for five questions or less [28].

### 2.8. Synthesis of Results

The food security programs were summarised in a tabular format and a narrative summary synthesises the key information which relates to the aims of this review. It is thought that by reviewing the literature and documenting effective food security initiatives worldwide, the lessons learned therein may provide guidance for future food security programs more broadly.

## 3. Results

### 3.1. Search Results

The database searches identified 3555 records, and 396 were identified from grey literature searching. After 1205 duplicates were removed, 2746 title and abstracts were screened, and 2397 records excluded. A total of 349 full text reports were assessed using the eligibility criteria, and of these, nine were included in this scoping review (see Figure 1).

### 3.2. Program Selection and Characteristics

Nine peer-reviewed publications were included in this scoping review [29,30,31,32,33,34,35,36,37] with no grey literature meeting the inclusion criteria (see Table 1). Five studies were from the United States of America [33,34,35,36,37] and four from Canada [29,30,31,32]. No studies from Australia or Aotearoa/New Zealand met the inclusion criteria. The included studies covered a 12-year time period between 2010 and 2022. The sample size of programs ranged from fewer than 100 in two publications [31,36] to over 1000 respondents in three publications [34,35,37]. Two reported either community [29,30] or household involvement [32,33] rather than individual participants. Three publications were program evaluations [29,30,35], two were cross-sectional [32,33], two were randomised trials [34,37], one a pre- and postcomparison [36], and one was ethnographic in design [31]. 

### 3.3. Program Aims

There were two studies that evaluated the Food Mail Program [29], later known as the Nutrition North Canada Program [30] which subsidised transportation costs of food to northern remote communities in Canada. The study by Timbler et al. [31] aimed to address First Nations community food insecurity with a Prison Garden Program whereby incarcerated men grew and donated produce to Tŝilhqot’in communities. The study by Blanchet et al. [32] was a 12-year First Nations food sovereignty intervention of salmon reintroduction in Syilx Okanagan Nation. Bersamin et al. [36] evaluated a Fish to School Program (Neqa Elicarvigmun) among Alaska Native peoples. Four interventions used voucher/food assistance programs [33,34,35,37]. Pindus et al. [33] aimed to understand participation and program operations of the FDPIR for American Indian and Alaska Natives, while Mucioki et al. [35] investigated opportunities and challenges of the FDPIR in achieving food security for the Karuk, Yurok, and Klamath tribe. The study by Gordon et al. [34] included two Indian Tribal Organisations (ITO) in their study (Cherokee and Chickasaw Nation), and the intervention group received benefits using WIC to assess the impacts on food security and child’s food consumption. Briefal et al. [37] assessed whether the Packed Promise intervention reduced food insecurity in 12 rural countries within Chickasaw Nation.

### 3.4. Measurement of Food Security and Diet

The approach to measure food security was reported in four studies [32,33,34,37]. Blanchet et al. [32] assessed food security by two methods: (1) an 18-item USDA adapted Household Food Security Survey Module and (2) Cultural food security questions related to traditional foods. Briefal et al. [37] also used the 18-item USDA Household Food Security Module (reference period of 30 days). Pindus et al. [33] used a six-item short form measure. Gordan et al. [34] used the USDA food security measure. Dietary assessment method was reported in three studies [32,34,36]. Blanchet et al. [32] and Gordon et al. [34] both used a food frequency questionnaire (FFQ). Bersamin et al. [36] used a single 24 h recall and biomarkers. Briefel et al. [37] conducted telephone surveys, but the dietary assessment was not reported.

### 3.5. Implementation Methods of the Interventions

Various intervention components were implemented including educational workshops or camps [31,32] highlighting the benefits of traditional diets [36], nutrition activities [29,30,33], distribution of recipes [31,33,37], nutrition education handouts [31,33,37], individual nutrition counselling [33], cooking classes, demonstrations or workshops [29,31,33], taste testing at grocery stores [29], serving locally caught traditional food in school cafeterias [36], community events celebrating traditional foods [36], food vouchers/boxes [33,34,35,37], subsidising the cost of transportation to facilitate access to healthy foods [29,30], and preparing nutritious and culturally-mediated dishes for free community lunches [31].

### 3.6. Community-Based Participatory Research

Three programs documented the use of community-based participatory research [31,32,36]. The study by Bersamin et al. [36] collaborated with a community working group (10 members) so that nutrition activities were designed to reflect Yup’ik worldviews, values, and traditional knowledge systems with evidence-based strategies for the Fish to School Program. The study by Timler et al. [31] described that their research was founded on the concept of community-based participatory research and decolonising research [22,23]. The study by Blanchet et al. [32] used decolonising research whereby the program was developed and implemented in partnership with Syilx community members. Leadership included Syilx Elders and cultural knowledge keepers, nation members, and technical staff. While two studies did not specifically mention that a participatory research approach was undertaken, tribal or community involvement was reported. The data gathered by Mucioki et al. [35] was part of a larger tribal food security project in collaboration with Karuk, Yurok, and Klamath Tribes in the Klamath River Basin. The study by Briefal et al. [37] had nutritionists working on the food boxes who were Chickasaw Nation tribe members. They communicated with Chickasaw families and ensured that the items selected for the food boxes were nutritious.

### 3.7. Program Outcome

The two studies that evaluated the Food Mail Program [29], later known as the Nutrition North Canada Program [30], reported mixed results. While stakeholder consultations reported that the program provides value to the community, key barriers exist, and the program was not shown to be fair and equitable across regions and communities. While benefits were reported for the men in the Prison Garden Program [31], the community described that receiving the produce had minimal impact on food security as the ongoing colonial contexts and food sovereignty needs to be considered. The community cooking workshops increased benefit by strengthening relationships and responsibilities among the community, the men in prison, and the sharing of food. The Syilx-led reintroduction of Okanagan Salmon intervention [32] reported that food sovereignty initiatives can increase access to and consumption of traditional food and enhance cultural food security. The Fish to School Program [36], a seven-month intervention, showed significant diet quality (4.57 times greater) and fish consumption improvements (0.16 times greater) for the intervention compared to the comparison community. The program increased the student’s connection to culture and supported food security promoting local food systems. The study by Pindus et al. [33] reported on the FDPIR program and illustrated how programs using nutrition assistance can co-exist with initiatives at a local level to meet nutritional needs of the community. The study by Mucioki et al. [35] also reported on the FDPIR and proposed a list of actionable policy recommendations to better support First Nations food security and food sovereignty. The intervention using the Summer Electronic Benefit Transfers for Children (SEBTC) by Gordon et al. in 2012 [34] showed that the group that received benefits had large and significant reductions in child food security and their dietary intake was composed of more healthful foods. The Packed Promise Intervention effectively delivered food boxes that were nutritious, but the intervention did not significantly reduce food insecurity for children at 12-months or 18-months; however, food insecurity was reduced for adults by three percentage points at 12-months [37].

### 3.8. Critical Appraisal

According to the QAT, three studies were assigned a high rating [32,35,36], two moderate [31,37], and four low [29,30,33,34] (see Supplementary Material Table S2). Most studies demonstrated First Nations governance so that the research benefited from the participants and communities, and the findings translated into sustained changes in policy and/or practice. Some studies responded to a need or priority in the community, had First Nations leadership, and demonstrated community engagement and consultation, with research guided by First Nations research paradigms and taking a strengths-based approach. Frequently lacking was the reporting on rights and agreements relating to existing and created cultural and intellectual property, local community protocols that were followed and respected, capacity strengthening as well as learning from each other through the research process.

## 4. Discussion

This scoping review considered programs addressing food security for First Nations peoples in Australia, Aotearoa/New Zealand, Canada, and the United States of America. It provides researchers, policy makers, and practitioners with an evidence base to co-design food security programs with First Nation Australian’s voices leading the way. All aspects of the review included First Nations Australian researchers, and we critically appraised the literature through a decolonisation lens sensitised by our personal lived experiences of food insecurity in colonial Australia.

All nine peer-reviewed publications identified were conducted in the United States of America or Canada. The programs most effective in improving food security for First Nations peoples were participatory in design, governed and led by community, reflecting their priorities and needs. Other effective elements of successful programs were those that integrated cultural knowledge and food systems to increase the accessibility and availability of cultural foods. Education components were important features of many programs. Food and transport subsidy programs were used for remote communities, but mixed results for effectiveness were observed. A number of programs addressed food security through the use of food or commodity boxes and while these constitute a significant source of food for many families and communities experiencing food insecurity, this form of intervention was seen to be enhanced by utilisation of interagency collaborations.

Community-based participatory research (described as co-design in Australia), where researchers and community stakeholder groups participate as equal collaborators throughout every stage of the research process, promotes the implementation of culturally appropriate, evidence-based interventions that enhance the translation of research findings for communities and policy change [38,39]. This scoping review identified three studies that specified the use of community-based participatory research to improve food security, with two out of the three receiving a high rating using the QAT. One being a farm-to-school multilevel intervention [36] and the other, a salmon restoration program [32]. The program that received a moderate score was founded on the principles of community-based participatory research, but some steps of the intervention lacked community input [31]. Interventions with low scores did not report the use of community-based participatory research and resulted in reduced program effectiveness. The critical importance of community-led and co-designed implementation and evaluation of programs for First Nations communities is well documented in Australia [40,41], this further holds true and is supported for studies in the other First Nations communities of the United States of America [42,43,44], Aotearoa/New Zealand [45,46], and Canada [47,48].

The integration of cultural knowledge increased program acceptability and uptake. For many First Nations peoples, food security requires food sovereignty which includes land and water rights. The food sovereignty intervention in Syilx Okanagan Nation of Canada by Blanchet et al. [32] was a multilevel community-led restoration program to increase the supply of their cultural food, being salmon. This intervention improved two pillars of food security: availability and accessibility. Furthermore, salmon access was linked with enhanced cultural food security, and restoration ensured preservation of Syilx culture. The farm-to-school multilevel intervention in two remote Alaska Native communities by Bersamin et al. [36] promoted local traditional food systems which supported food security using traditional foods (specifically fish). Both the consumption of fish and overall diet quality significantly improved in the intervention compared to the comparison community. These findings indicate the lesson that knowledge and skill transmission and the sharing of the fish amongst the community enables cultural food security and true food sovereignty. This was highlighted in Australia where a media article reported a cultural fishing permit granted so that traditional net-fishing can be practiced which enhances community connection, culture, and wellbeing [49]. However, to promote such restoration programs that enhance food sovereignty for First Nation communities in Australia, legislative action regarding the rights of water sovereignty requires attention to prevent the criminalisation of First Nations peoples continuing their cultural practices of fishing and food procurement [50].

Education components such as nutrition education workshops or camps, cooking classes, information handouts, and recipe distribution were common. By implementing fishing camps, Blanchet et al. [32] supported First Nations traditional knowledge and skill transmission. The multilevel intervention by Bersamin et al. [36] integrated activities spanning three settings (cafeteria, classroom, and community) which increased students’ knowledge on traditional food systems and their connection to culture. These findings align with Australian research that recommends multistrategic and multilevel approaches to complex issues such as food security [51]. Ensuring local culture is embedded in health promotion programs will help to address challenges in program adoption and sustainability, whilst maintaining cultural integrity [52]. When culturally appropriate cooking workshops were incorporated into the interventions, this reportedly strengthened relationships and nutritional knowledge [31] and led to changes in cooking practices or eating habits [33]. The importance of funding and resource allocation should be acknowledged as a barrier to ongoing program implementation [32]. This is a common theme across all First Nations research, and it is well known that community-based programs in Australia experience barriers, including adequate funding for end-to-end holistic programs that are evaluated and sustainable [53], and the lesson is to provide for long-term funding.

Increasing the availability and access to healthy food where First Nations people live is important to enhance food security. One program aimed to increase the access to healthy foods, specifically in remote areas, through subsidies for food transportation [29,30]. While mixed results were observed, lessons can be learned from the concerns raised by stakeholders and the community regarding the logistical aspects of accessing food drop-off locations, the absence of culturally suitable or accepted foods on the eligibility list, and that many community members did not have a credit card, which consequently hindered the ability to place personal food orders. In remote regions of Australia, major supermarkets are not easily accessible to First Nations peoples and the cost for nutritious foods is substantially higher in comparison with differences as much as AUD 221 for the price of the same food basket [54]. Similar to the Food Mail Program [29,30], higher food prices are likely due to the costs involved in transportation such as fuel usage, but also in Australia, many remote communities have one store, and the limited competition can lead to price gouging [55]. A report from 2023 in a rural town in NSW showed that many community members struggle with affordability 71% (limited funds), availability 63% (limited supermarkets/food stores), accessibility 47% (issues with transportation to purchase food), and safety 24% (issues with food safety, including preparation) [9]. These programs highlighted the absence of an integrated approach to systemic issues of transportation, cost, cultural food availability, stores and pricing, market competition, and feedback from First Nations peoples about the food system.

Nutrition assistance programs and use of food boxes was another intervention method aimed at increasing access to healthy food for First Nations communities [33,34,35,37]. To address the concern around children’s food insecurity in summer months, Gordon et al. [34] assessed the use and impact of WIC or SNAP and a reduction in very low food security was reported. Mucioki et al. [35] and Pindus et al. [33] reported on the FDPIR, which was established to specifically offer food assistance to rural First Nations communities. Although concerns have been raised around the impact on government programs on First Nations traditional ways [56], the monthly food boxes were shown to be a major food supply for many food-insecure families and communities. A key lesson is that a more “food sovereign” approach and integrating traditional foods may enhance food security and effectively address the needs of communities [33,35].

Utilising interagency collaborations and linking with existing institutional structures is important [42]. Pindus et al. [33] discussed how multisector partnerships facilitated access to healthy food options through initiatives such as transportation services, food delivery in community, expansion of farmer’s markets, and encouraging home gardening. Partnerships were formed between tribal and country, State or Federal Government, philanthropic foundations, community organisations, schools, farmers, and health centres which enhanced capacity on Indian country and improved food access. This provides a practical example of how federal nutrition assistance programs can co-exist with local initiatives to ensure accessibility and availability of healthy food choices. While the United States of America has a federal nutrition assistance program to income-eligible households, no federal nutrition assistance exists in Australia. Emergency food relief is supplied through not-for-profit charity organisation, but First Nations Australians often experience stigma and shame when accessing these services [57]. Developing interagency collaborations to provide food relief may provide a more culturally appropriate and acceptable process of leveraging existing structures. Given an additional burden to First Nations families and communities when accessing food relief was transportation [29], subsidising food “drop-off” or fostering interagency collaboration with community transport services could mitigate transportation challenges.

While the focus of this review was to provide Australia with guidance on lessons learned from international food security programs, it also provided a platform in which other countries can apply these findings. For instance, Aeotearoa/New Zealand had a lack of evaluation of food security programs. As recommended for Australia, each of the key successful elements can be adapted for Māori peoples in Aeotearoa/New Zealand, in consideration of their social and political landscape. The programs from the United States of America mainly reported on food assistance and, given that Canada lacks a federal food relief program, this is something which could be implemented as it supplied short-term emergency food relief. Programs in Canada, however, used food transport subsidies to increase the access to healthy foods in remote communities, with subsidies lacking in the United States of America. While mixed results were reported, it was evident that barriers to uptake were due to suboptimal community consultation.

This review provides policy makers with lessons to adapt for First Nation Australians for a multifaceted food security program premised on key successful elements of participatory research, integration of cultural knowledge, education, food and transport subsidy, food or community boxes, and interagency collaboration. Despite the rigorous and novel approach, this review is not without its limitations. The authors are aware of active food security initiatives in Australian First Nations communities, but they lack reporting and evaluation which resulted in the limited discussion and documentation around program effectiveness in this review. Program evaluation is needed to prove effectiveness to inform nutrition policy and practice. Programs were excluded if they indirectly addressed food (in)security, an example being a fruit and vegetable intervention [58]. The QAT was designed for an Australian context and although all countries share similar colonial history, the tool has not been validated for use across First Nations peoples of Canada and the United States of America. Furthermore, the QAT was developed in 2018 and most of the programs with low scores were published prior to the development of this tool or focused on learnings from nutrition assistance programs. Therefore, this should not be viewed as discrediting previous research, but rather as an opportunity to highlight the documentation of tools that reflect First Nations worldviews and research paradigms.

## 5. Conclusions

This review identified important lessons learned from food security programs in Canada and the United States of America and highlighted the lack of evaluation of food security programs in Australia and Aotearoa/New Zealand. The lessons learned from food security initiatives worldwide may provide guidance for food security programs in Australia. It is clear that future programs in Australia need to use community-based participatory research and have strong community governance. The integration of cultural knowledge and food systems through restoration programs may enhance food sovereignty for First Nations Australian communities, but legislative action regarding the rights of water sovereignty requires attention. Education that integrates activities spanning many settings may increase knowledge on traditional food systems and connection to culture. Nutrition assistance requires a more “food sovereign” approach and integrating traditional foods may enhance food security and effectively address the needs of communities. Finally, the use of interagency collaborations for food relief may provide a more culturally appropriate and acceptable process of leveraging existing structures. Given an additional burden to First Nations Australian families and communities when accessing food relief is transportation, subsidising food “drop-off” or fostering interagency collaboration with community transport services could mitigate transportation challenges. Findings showed that while it is important to address short-term emergency food relief, the aim should be sustainable food security through a longer-term system and policy change underpinned by co-designed research and evaluation.

## Figures and Tables

**Table 1:** Programs for addressing food security for First Nations people of Australia, Aotearoa/New Zealand, Canada, and the United States of America.

First Author, Year, Ref	Country of Origin	Aims */Purpose	Study Population and Sample Size	Methodology/Methods	Intervention	Key Findings
Majid, 2010 [29]	Canada. (northern remote regions).	To increase access to healthy foods for those in isolated communities in the north by subsidising the cost of food transport.	Northern communities (*n* = 135) were eligible to receive subsidised food. Isolation for short periods of time (ineligible).	Program evaluation. Qualitative (stakeholder consultations by internal/external consultants and an independent appointee from the Canadian Government) Analysis not described.	Food Mail Program. (1) orders placed; (2) items transported from wholesalers and flown by regional carriers to distribution points; (3) delivery to final destination between 3 and 7 days depending on recipient location.	Program impact: mixed results Very few First Nations communities familiar with the program/leadership. Never taken part in the program (at least *n* = 30 eligible communities). Program growth over five years with more healthy food being consumed (compared to previous years). Price: surveyed participants questioned if retailers were passing on the subsidy. Place: collecting the orders proved difficult for both retailers and individual consumers (limited transport and dangerous weather conditions). No access to credit card to place orders. Produce: foods eligible were criticised and reported to be not cultural/stable foods. Spoilage of perishable food.
Galloway, 2017 [30]	Canada. (remote northern regions).	To provide an independent and comprehensive evaluation of the Nutrition North Canada program.	Northern communities (*n* = 128).	Program evaluation. Mixed methods employing a modified conceptual framework. Focus on program performance/equity of outcomes. Independent, comprehensive evaluation conducted. Data extracted from program documents (fiscal and food cost reports), compliance reports from retailers and program audits. Program performance measurement strategy used to understand if the subsidy is meeting its objectives (comprehensive and equitable across regions/communities) Descriptive statistics to compare results among provinces, territories, and communities.	Nutrition North Canada Program (replaced Food Mail Program). Retail subsidy to provide reliable and affordable access to fresh/nutritious food. Operates within a context of food insecurity (severe) within a budget of CAD 60 million per year. Subsidy is paid to retailers directly who sell the eligible foods in local stores. The retailers participate in nutrition education activities. Program promoted on cash register, receipts, signage, and displays.	The program did not ensure fair and equitable access to fresh/nutritious food across regions/communities. The program did not respond to a range of concerns (eligibility of community/foods, rates of subsidies). Retailer accountability raised by community members, critics, the Auditor General of Canada. Program’s own Advisory Board Program lacked adequate evidence base.
Timler, 2019 [31]	Canada. Prisoners and Tŝilhqot’in community members in British Columbia. (rural and remote).	A prison garden program to address inmate rehabilitation and First Nations community food insecurity by supporting incarcerated men to grow and subsequently donate organic produce to rural and remote First Nations communities.	Interviews conducted (*n* = 10; Tsilhqot’in community members); prison garden workers (*n* = 10; men); program stakeholders (*n* = 5).	Design: Ethnographic Qualitative (data collected via interviews and participant observation). Conducted within the context of a broader prison-community partnership research program. Purposive sampling. Community-based participatory research. Decolonising and ethical research undertaken. Iterative thematic analysis to explore descriptive patterns and develop interpretive themes. Interviews coded using NVivo.	Prison-community vegetable distribution process. The prison garden operates within the wider colonial context. The garden was founded in 2004 with the intention of reducing idle time by providing meaningful work for federally incarcerated men through the growing and donating of organic produce, intended to address food insecurity in surrounding communities. Vegetables donated to local food banks, school lunch programs, homeless shelters, and First Nations communities. Cooking workshops conducted within three communities.	The benefits for the men working were layered, deepening over the time, and influenced by personal histories and contexts. There is potential that the prison garden may increase community engagement and recognise strengths of the community. The benefits for the community receiving the produce were minimal as the distribution of vegetables aimed at addressing food security and was thus unable to redress the ongoing colonial context that impedes access to meaningful and culturally mediated foods and foodways. Community cooking workshops increased benefit by strengthening relationships and responsibilities among the communities, the men in prison, and the foods they share.
Blanchet, 2022 [32]	Canada. Syilx Okanagan Nation.	To describe the reach of the Syilx-led reintroduction of Okanagan Sockeye salmon intervention and assess its impact on Syilx households’ income-related and cultural food security status.	All Syilx communities were invited to participate (*n* = 7) and *(n* = 3) agreed. Completed interviews (*n* = 265 households). Participation rate (82.8%).	Cross-sectional interviewer survey. Community-based participatory research. Decolonising health promotion framework used. Randomly selected: More than (*n* = 250 households) in the community. All selected: Less than (*n* = 250 households) in the community. Food security measurement: (1) adapted 18-item USDA Household Food Security Survey Module; (2) questions on cultural food security (worried about running out of traditional foods) Dietary assessment: traditional food consumption evaluated using a traditional FFQ (adapted by the participating community). Frequencies and means, Spearman correlation, v2 tests, Fisher’s exact tests, and *t*-tests. Analysis done in SAS.	12-year Syilx-led Sockeye Salmon Reintroduction (First Nations food sovereignty intervention). Hatchery supplementation for sockeye salmon restoration. Education components included that allowed children to raise salmon fry in class/release at a yearly Ceremony. Fishing camps organised in the first year of the intervention (knowledge and skill transmission).	Food insecurity was prevalent; income (47%) and cultural related (63%). 21% would often or sometimes (42%) worry that traditional food would run out. Benefits of intervention: households received salmon from a community program (80%), harvested more salmon (28%), accessed store-bought salmon (13%), or equipment/other resources to fish (9%). Households that accessed salmon were less frequently worried that traditional food would run out and reported that traditional food was important in ensuring their household had enough food compared to households with limited access to salmon. First Nations food sovereignty initiatives increase access to and consumption of traditional foods and supports cultural food security.
Pindus, 2019 [33]	America. American Indian and Alaska Natives.	To develop a national profile of FDPIR participation and an understanding of program operations.	Households of all ages (*n* = 1053). Completed interviews (*n* = 849). Response rate (83%). Survey response: consistently high across the programs (range 69 to 95%).	Mixed methods, culturally responsive study design. Cross-sectional survey with qualitative interviews with trial leaders/extensive outreach with certain tribes and FDPIR sites. Sampling strategy had two stages. Primary data collection and analysis of secondary data in order to establish a national profile of participation in FDPIR and have an understanding of the operations of the program. 30 min survey administered with the FDPIR applicants (in person or over the phone). Food security measurement: 6 item short form measure (used by the Economic Research Service). Site visits: obtain information about nutrition education and health promotion. Researchers interviewed FDPIR directors and staff, tribal leaders, and other community members. Food access: examined using the research atlas (produced by Economic Research Service). No description of analysis.	A study of the FDPIR program (program administered at the federal level). Supplemental food package program. Monthly food packages provided to income eligible households living on Indian reservations/tribal lands/Alaskan Native villages and American Indians residing in designated areas near reservations or in the state of Oklahoma. Administration of the program locally: ITO/state government agencies.	44% households (food secure); 34% of households (low food security, reduced food quality, variety, or desirability of diet but not reduced intake); 22% of households (very low food security)/disrupted eating patterns and reduced intake. Primary food source for 38% of households, contributing 81 to 100% of food supply. Multisector partnerships facilitated access to healthy food options by providing transportation, offering food delivery at the community level, encouraging home gardening, and expanding farmer’s markets (partnerships illustrate how federal nutrition assistance programs can co-exist with initiatives at a local level). Promotes healthy eating through nutrition education activities using a variety of federal, state, and local tribal resources (nutrition education suggested as a worthwhile investment).
Gordon, 2017 [34]	America. 8 states and 2 ITOs (Cherokee Nation, Chickasaw Nation)	To inform food assistance policy and describe how demonstrations using WIC and the SNAP models differed in benefit take-up and impacts on food security and children’s food consumption.	Grantees (*n* = 8 states); (*n* = 2 ITO) selected school districts for many low-income children. The evaluation sample (*n* = 42,000 households). Response rate: 73% (spring); 80% (summer). Summer Electronic Benefit Transfers for Children (SEBTC); WIC model grantees—Cherokee Nation/Chickasaw Nation: evaluation subsample (*n* = 17,000).	Mixed-methods randomised trial. Sites delivered SEBTC using SNAP or WIC, EBF. Households randomly assigned to benefit group or control. Household interviews: baseline (during spring) and at 30 days to measure effects of SEBTC on child food security and food intake (during summer). Food security measurement: USDA food security scale. Dietary assessment: FFQ used in the NHANES Dietary Screener Questionnaire. Mean outcome differences between the benefit and control group measured impact (adjustments for household characteristics) using a weighted linear regression.	Intervention: households received benefits from grantees of USD 60 per child during summer using SNAP or WIC EBT systems. Most foods covered by SNAP-model benefits/specific foods covered in WIC-model benefits. Outcome: food security and food consumption for children.	Large reductions (very low food security) among children for the benefit group. SEBTC benefits had a positive impact on children’s food consumption from healthy foods. Larger for WIC-model than SNAP-model benefits.
Mucioki, 2018 [35]	America. Indian Reservations, Klamath Basin, Karuk tribe, Yurok tribe, Klamath tribe.	To investigate opportunities and challenges of the FDPIR to achieve food security as well as the extent to which integration of traditional foods can enhance Native American food security, food sovereignty and wellbeing.	Klamath, Karuk, and Yurok tribes. Karuk people (*n* = 6115), Klamath people (*n* = 4413), Yurok people (*n* = 6504) included. Key informant interviews (*n* = 9 interviews). Focus groups (*n* = 20) with a total of (*n* = 128 Native American participants). Tribes: Karuk (5 groups), Yurok (8 groups) and Klamath (7 groups). Average of (*n* = 7) per group. Household survey (*n* = 3851 distributed, *n* = 707 complete) to assess food access, utilization of food systems and food assistance including FDPIR. In depth interviews (*n* = 14) with tribal cultural practitioners.	Program evaluation using mixed methods (consisted of key informant interviews of FDPIR program managers, administrators, advocates (national and local level), focus groups, and a household survey co-created with tribal partners). Tribal cultural practitioners involved interviews for insight into the local experience of FDPIR and complemented food assistance related survey questions. Interviews and focus groups coded using NVivo. Analysis of quantitative data from surveys (descriptive statistics and two-tailed Fisher’s exact test). Stata used to establish significance of key outcomes.	Food costs: USD 57 per participant/M (USD 1.90//day). USDA purchased and shipped FDPIR foods. Logistics (e.g., ordering, storing, distribution of food); determining eligibility; nutrition education to FDPIR clients by ITOs (*n* = 120) and state agencies (*n* = 3) (serving 276 tribes, pueblos, or nations).	60% reliance on food assistance. Commodity boxes needed for food security (low-income Native American households). Monthly commodity boxes stretched income to cover other monthly expenses. Integrate traditional foods requested. Households continue to source traditional foods (hunt and fish). Drop-off services helped those with limited access/costs associated with transportation. All FDPIR managers (*n* = 3) reported ongoing problems with receiving spoiled produce.
Bersamin, 2019 [36]	America. Alaska Native (rural and remote)	To evaluate the preliminary efficacy of a school-based intervention on diet quality, fish intake, and attitudes and beliefs around traditional foods.	Middle school/high school students from (*n* = 2 communities). (*n* = 76 participants), time points (T1 baseline, T2 4 M; and T3 9 M). Yup’ik (99% identified); followed a Yup’ik way of life (32%). Attrition rate: 27% (intervention) 26% (control).	Quantitative pre-/postcomparison design. Community-based participatory research. Consideration to the sample size/contamination between schools. Dietary assessment: Single 24 hr recall and biomarkers to measure fish intake. Descriptive statistics and multilevel analyses conducted in HLM.	“Fish to School Program” (Neqa Elicarvigmun) Overarching intervention framework: SCT combined with First Nations traditional knowledge. School-based, theoretical framework multilevel intervention. Intervention included activities in (1) cafeteria (salmon offered weekly for lunch); (2) classroom (cultural lessons, benefits of traditional diets, specifically fish; (3) community (intergenerational events which celebrated traditional foods which linked to school activities). Outcomes: Intake of fish, diet quality and attitudes/beliefs of traditional foods.	Strengths-based approach. Reconnected students to culture and their traditional food system. Benefit to student’s diet quality (4.57 times greater in the intervention group; *p* < 0.05). Increased fish intake (0.16 times greater in the intervention group; *p* < 0.05)
Briefel, 2021 [37]	America. Oklahoma, Chickasaw Nation Territory (12 rural countries).	To determine if the Packed Promise intervention reduces food insecurity among low-income households with children eligible for free school meals.	Low-income households (with children older than 4 years). Schools (*n* = 115). School districts (*n* = 40), randomly assigned to a treatment or control district (*n* = 20 each). A total (*n* = 4750 households) actively consented and randomised to participate in the evaluation (some determined ineligible at a later date) Survey response rates: 62% (baseline); 62% (follow-up 1); 61% (follow-up 2).	Quantitative cluster randomised control trial. Data collection: baseline (*n* = 2859); 2 follow-ups (*n* = 2852; *n* = 2790). Study design: random sample stratified by school district. Food security measurement: Standardised 18-item US Household Food Security Module (30-day reference period). Dietary assessment: telephone surveys (30 min) on food consumption (child) and food supply within the household. Sample weights applied (as a complex design/adjustment for non-responders). Standard errors accounted for clustering and stratification of households. Differences between the treatment and control groups estimated by regression models (controlling for baseline characteristics) in STATA.	Packed Promise intervention. Households selected shelf-stable nutritious food boxes (5 varieties prepared by tribal nutritionists who communicated with Chickasaw families). Cost of food in each box (USD 38) and households given USD 15 to purchase fruit and vegetables. Nutrition education handouts included. 25 M intervention. Outcome: Child food insecurity (primary). Adult/household food security and food expenditures (secondary).	Successful delivery of nutritious food boxes to children of low-income households. Did not significantly reduce child food insecurity at 12 or 18 M. Food insecurity (adults) reduced by 3% points at 12 M, but not at 18 M. Intervention led to a USD 27 (12 M) and USD 16 decline (18 M) in median household monthly out of pocket food expenditures. Treatment households (97%) ordered at least one food box. Participation rate in monthly orders had a mean of 61% during the course of the intervention (project admin data).

Electronic Benefit Transfers System (EBF); food frequency questionnaire (FFQ); Food Distribution Program on Indian Reservations (FDPIR); Indian Tribal Organisations (ITO); Months (M); National Health and Nutrition Examination Survey (NHANES); Special Supplemental Nutrition Program for Women, Infants, and Children (WIC); Summer Electronic Benefit Transfers for Children (SEBTC); Supplemental Nutrition Assistance Program (SNAP); Social Cognitive Theory (SCT); United States Department of Agriculture (USDA). * Language used in Aims column comes directly from the original paper.

## Data Availability

Not applicable.

## Supplementary Materials

The following supporting information can be downloaded at https://www.mdpi.com/article/10.3390/nu15143127/s1; Table S1: MEDLINE search strategy; Table S2: The 2018 SAHMRI CREATE First Nations results of peer-reviewed articles.
