# Supplementary material for: Programs Addressing Food Security for First Nations Peoples: A Scoping Review
Source: Nutrients. 2023 Jul 13;15(14):3127. doi: 10.3390/nu15143127 (PMC10384335; doi:10.3390/nu15143127)
Supplement: Supplementary file 1 [file nutrients-15-03127-s001.zip › nutrients-2491512-supplementary.pdf]

Supplementary Table S1. Medline Search Strategy.

#	Query	Results
1	Indigenous Peoples/	1,020
2	"Native Hawaiian or Other Pacific Islander"/	11,864
3	Health Services, Indigenous/	3,913
4	(Aborigin* or Indigen* or First People* or First Nation* or 1st nation* or Koori*).tw.	55,580
5	or/1-4	61,230
6	Australia/	120,407
7	New South Wales/	13,879
8	(Australia* or New South Wales or NSW or N S W).tw.	167,560
9	(Australia* or New South Wales or NSW or N S W).in.	712,016
10	or/6-9	792,893
11	exp Food Insecurity/	1,062
12	exp Food Security/	464
13	Food Storage/	4,119
14	Cooking/	13,911
15	Hunger/	5,904
16	exp Food Supply/	16,218
17	Food Assistance/	1,666
18	Malnutrition/	17,703
19	Starvation/	10,146
20	nutritional status/	51,864
21	exp Nutritional Requirements/	22,074
22	(Food* adj4 (secur* or insecur* or sufficien* or insufficien* or access* or accept* or availab* or acquir* or supply or supplies or usage or utilisation or utilisation or stability or stable or adequa* or provide or provision or production or share or sharing or good or choice* or choos* or resource* or price* or cost* or quantit* or store* or storage or knowledg* or prepar* or system* or healthy or bank* or pantr* or relief* or hamper* or stamp* or assistance or protect* or land* or sea or seas or water* or river* or lake* or stream* or spring* or ground or borehole or billabong or tradition* or nontradition* or household* or communit* or ancest* or sovereign* or vulnerab* or socioeconomic* or economic* or equity or afford* or poverty or educat* or train* or finance* or income or fund* or employ* or unemploy* or job* or vocation* or work* or career* or occupation* or coloni?ation or colonial or precoloni* or settlement* or setting or relocate* or mission* or reserve* or oppression or cultur* or holistic or wellbeing or spiritual or physical or social or emotional or ecological or knowing or	234,673

#	Query	Results
	yarn* or intake* or diet* or nutriti* or processed or ultraprocessed or discretionary or covid* or corona* or SARSCoV2 or behavio?r or habit* or determinant* or risk* or commercial or shop* or marketing or industr* or advertis* or regional or remote or rural or urban)).tw.	
23	(Nutriti* adj4 (secur* or insecur* or sufficien* or insufficien* or access* or availab* or acquir* or supply or supplies or usage or utilisation or utilisation or stability or stable or adequa* or provide or provision or share or sharing or good or choice* or choos* or resource* or price* or cost* or quantit* or store* or storage or knowledg* or prepar* or system* or healthy or assistance or protect* or land* or sea or seas or water* or river* or lake* or stream* or spring* or ground or borehole or billabong or tradition* or nontradition* or household* or communit* or ancest* or sovereign* or vulnerab* or socioeconomic* or economic* or equity or afford* or poverty or educat* or train* or finance* or income or fund* or employ* or unemploy* or job* or vocation* or work* or career* or occupation* or coloni?ation or colonial or precoloni* or settlement* or setting or relocate* or mission* or reserve* or oppression or cultur* or holistic or wellbeing or spiritual or physical or social or emotional or ecological or knowing or yarn* or intake* or diet* or food* or status or requirement* or need* or processed or ultraprocessed or discretionary or covid* or corona* or SARSCoV2 or behavio?r or habit* or determinant* or risk* or commercial or shop* or marketing or industr* or advertis* or regional or remote or rural or urban)).tw.	129,626
24	(Diet* adj4 (sufficien* or insufficien* or access* or availab* or acquir* or supply or supplies or adequa* or provide or provision or share or sharing or good or choice* or choos* or resource* or cost* or knowledg* or prepar* or healthy or assistance or protect* or land* or sea or seas or water* or river* or lake* or stream* or spring* or ground or borehole or billabong or tradition* or nontradition* or household* or communit* or ancest* or socioeconomic* or economic* or equity or afford* or poverty or educat* or train* or finance* or income or fund* or employ* or unemploy* or job* or vocation* or work* or career* or occupation* or coloni?ation or colonial or precoloni* or settlement* or setting or relocate* or mission* or reserve* or oppression or cultur* or holistic or wellbeing or spiritual or physical or social or emotional or ecological or knowing or yarn* or intake* or nutriti* or food* or status or requirement* or need* or processed or ultraprocessed or discretionary or covid* or corona* or SARSCoV2 or behavio?r or habit* or determinant* or risk* or commercial or shop* or marketing or industry or advertis* or regional or remote or rural or urban)).tw.	181,291
25	(Tucker or grub*).tw.	5,100
26	(hunger* or hungry or malnutrition or malnourish* or starv*).tw.	103,140
27	Cooking facilit*.tw.	41
28	or/11-27	589,814
29	5 and 10 and 28	639
30	limit 29 to yr="2010 -Current"	427

Supplementary Table S2. The 2018 SAHMRI CREATE First Nations results of peer-reviewed articles.

First Author, Reference	Indigenous Governance				Respect for Cultural and Intellectual Property				Capacity Building			Beneficial Outcomes			Overall Assessment
	Q1	Q2	Q3	Q4	Q5	Q6	Q7	Q8	Q9	Q10	Q11	Q12	Q13	Q14	
Majid	N	N	N	Y	U	U	N	N	N	U	Y	P	N	N	Low
Galloway	P	N	Y	U	N	N	N	N	N	N	P	N	N	N	Low
Timler	U	U	U	P	U	U	U	P	Y	P	U	P	P	U	Moderate
Blanchet	Y	Y	Y	Y	Y	Y	Y	Y	Y	Y	U	Y	Y	U	High
Pindus	U	P	N	N	U	U	U	U	U	N	P	N	P	N	Low
Gordon	N	N	N	U	N	N	N	N	U	U	P	P	N	N	Low
Mucioki	Y	Y	Y	Y	Y	U	U	Y	Y	Y	Y	Y	Y	Y	High
Bersamin	U	Y	Y	P	Y	Y	Y	Y	Y	Y	Y	Y	Y	Y	High
Briefal	Y	Y	Y	Y	U	U	U	N	U	U	P	Y	N	N	Moderate

Y = Yes, P = Partially, U = Unclear, N = No explicit statements in the body of the text to provide evidence for each question below

1. Did the research respond to a need or priority determined by the community?
2. Was community consultation and engagement appropriately inclusive?
3. Did the research have First Nations research leadership?
4. Did the research have First Nations governance?
5. Were local community protocols respected and followed?
6. Did the researchers negotiate agreements in regards to rights of access to existing First Nations peoples' intellectual and cultural property?
7. Did the researchers negotiate agreements to protect First Nations peoples' ownership of intellectual and cultural property created through the research?
8. Did First Nations peoples and communities have control over the collection and management of research materials?
9. Was the research guided by an Indigenous research paradigm?
10. Does the research take a strengths-based approach, acknowledging and moving beyond practices that have harmed First Nations in the past?
11. Did the researchers plan to and translate the findings into sustainable changes in policy and/or practice?
12. Did the research benefit the participants and First Nations communities?
13. Did the research demonstrate capacity strengthening for First Nations individuals?
14. Did everyone involved in the research have opportunities to learn from each other?
